# Overcoming Biological Barriers: Importance of Membrane Transporters in Homeostasis, Disease, and Disease Treatment 2.0

**DOI:** 10.3390/ijms25105521

**Published:** 2024-05-18

**Authors:** Giuliano Ciarimboli

**Affiliations:** Experimental Nephrology, Department of Internal Medicine D, University Hospital Münster, 48149 Münster, Germany; gciari@uni-muenster.de

This editorial summarizes the seven scientific papers published in the Special Issue “Overcoming Biological Barriers: Importance of Membrane Transporters in Homeostasis, Disease, and Disease Treatment 2.0” of the International Journal of Molecular Sciences. This Special Issue presents comprehensive insights into transporter research. Transporters are pivotal in facilitating the passage of signal molecules, nutrients, metabolites, xenobiotics, and drugs across biological membranes, thus crucially contributing to maintaining homeostasis and aiding in drug management. Alterations in transporter functionality can disrupt homeostasis, trigger diseases, or influence the effectiveness of drug therapies. The objective of this Special Issue is to gather the latest findings on transporters, emphasizing their roles in various functions, regulatory mechanisms, pathological implications, and significance in both therapeutic outcomes and undesired adverse reactions. In addition to three reviews, the readers will find four original research works focusing on specific aspects of transporter physiology, pathophysiology, and pharmacology.

The review “Renal Organic Anion Transporters 1 and 3 In Vitro: Gone but Not Forgotten” by Caetano-Pinto and Stahl focuses on renal organic anion transporters, an important system mediating renal secretion of organic anions [1]. Organic anions are endogenous compounds such as uric acid [2], and exogenous compounds such as drugs and their metabolites and are part of the remote sensing and signaling system [3]. Considering that the expression of organic anion transporters (OATs) is lost in several renal cell lines, this review summarizes the mechanisms that regulate the expression and activity of OATs and presents the physiological changes that may cause the loss of these transporters in cell cultures.

In the review “A Role of Sodium-Glucose Co-Transporter 2 in Cardiorenal Anemia Iron Deficiency Syndrome”, Motoaki Sano describes the roles of sodium-glucose co-transporter 2 (SGLT2) in the regulation of energy metabolism, blood pressure, erythropoiesis, iron bioavailability, and inflammation in diabetes, heart failure, and renal disease. This is of particular importance since SGLT2 inhibitors seem to improve not only glycemic control in diabetes but also cardiorenal anemia iron deficiency syndrome [4].

Finally, the review “Role of the Sodium-Dependent Organic Anion Transporter (SOAT/*SLC10A6*) in Physiology and Pathophysiology” by Wannowius et al. summarizes the knowledge available of the sodium-dependent organic anion transporter (SOAT, gene symbol *SLC10A6*), a transporter from the SLC10 family [5]. This review presents information on SOAT expression and function, effects of its inhibition, and its role in steroid synthesis.

In the paper “Interactions of the Anti-SARS-CoV-2 Agents Molnupiravir and Nirmatrelvir/Paxlovid with Human Drug Transporters”, Bakos et al. investigated the interaction of these small molecules used in the treatment of COVID-19 [6,7] with drug transporters. Interestingly, greater interactions with drug transporters were observed using Nirmatrelvir and Ritonavir in combination.

In dopaminergic neurons, the dopamine transporter (DAT) mediates the release and reuptake of dopamine from the synaptic cleft. DAT function has been related to the development of neuropsychiatric diseases such as parkinsonism or attention deficit hyperactive disorder (ADHD) [8,9] and is also an important target molecule for amphetamine action. In the manuscript “Long-Lasting Epigenetic Changes in the Dopamine Transporter in Adult Animals Exposed to Amphetamine during Embryogenesis: Investigating Behavioral Effects”, Ke et al.’s use of the nematode *C. elegans* showed that chronic embryonic amphetamine exposition provoked epigenetic modification of DAT-1, which changed its expression.

Another important transporter for neurotransmitters is the plasma membrane monoamine transporter (PMAT, *Slc29a4*), which can mediate the passage through the plasma membrane of dopamine, serotonin, and norepinephrine or histamine [10]. In the paper “Heterotypic Stressors Unmask Behavioral Influences of PMAT Deficiency in Mice”, Weber et al. showed that PMAT function contributes to the determination of behavioral and physiological heterotypic stress responses in mice.

Steinbüchel et al.’s work, “Regulation of Transporters for Organic Cations by High Glucose”, focused on transporters for organic cations, which are highly expressed in secretory organs such as the liver and the kidneys [11,12]. They showed that their function can be stimulated by high glucose concentrations (16.7 mM). This stimulation seems to be associated with an increase in the expression of transporters in the plasma membrane and may be mediated by the mechanistic target of rapamycin (mTOR) kinase.

In conclusion, this Special Issue helps to delineate the physiological, pathophysiological, and pharmacological importance of plasma membrane transporters and will hopefully stimulate and help researchers to plan and conduct new studies.

The Guest Editor of this Special Issue would like to thank all the contributors, reviewers, and the Editorial Team of the International Journal of Molecular Sciences for their contributions, which made this work possible.
